# Genetically targeted chemical assembly of polymers specifically localized extracellularly to surface membranes of living neurons

**DOI:** 10.1126/sciadv.adi1870

**Published:** 2023-08-09

**Authors:** Anqi Zhang, Kang Yong Loh, Chandan S. Kadur, Lukas Michalek, Jiayi Dou, Charu Ramakrishnan, Zhenan Bao, Karl Deisseroth

**Affiliations:** ^1^ Department of Chemical Engineering, Stanford University, Stanford, CA 94305, USA.; ^2^ Department of Bioengineering, Stanford University, Stanford, CA 94305, USA.; ^3^ Department of Chemistry, Stanford Chemistry, Engineering and Medicine for Human Health (ChEM-H), Stanford University, Stanford, CA 94305, USA.; ^4^ CNC Program, School of Medicine, Stanford University, Stanford, CA 94305, USA.; ^5^ Department of Psychiatry and Behavioral Sciences, Stanford University, Stanford, CA 94305, USA.; ^6^ Howard Hughes Medical Institute, Stanford University, Stanford, CA 94305, USA.

## Abstract

Multicellular biological systems, particularly living neural networks, exhibit highly complex organization properties that pose difficulties for building cell-specific biocompatible interfaces. We previously developed an approach to genetically program cells to assemble structures that modify electrical properties of neurons in situ, opening up the possibility of building minimally invasive cell-specific structures and interfaces. However, the efficiency and biocompatibility of this approach were challenged by limited membrane targeting of the constructed materials. Here, we design a method for highly localized expression of enzymes targeted to the plasma membrane of primary neurons, with minimal intracellular retention. Next, we show that polymers synthesized in situ by this approach form dense extracellular clusters selectively on the targeted cell membrane and that neurons remain viable after polymerization. Last, we show generalizability of this method across a range of design strategies. This platform can be readily extended to incorporate a broad diversity of materials onto specific cell membranes within tissues and may further enable next-generation biological interfaces.

## Teaser

Genetically engineered neurons express membrane-bound enzymes that can catalyze oxidative polymerization on the cell surface.

## INTRODUCTION

Multicellular biological systems display highly complex structural and organizational properties, posing challenges for investigators seeking to achieve cell-specific electrical interfaces without inducing damage. For example, the human brain contains ~86 billion neurons with more than 100 trillion synaptic connections crucial for neuronal communication, but despite decades of effort in shrinking bioelectronic devices to the nanometer scale and scaling up the fabrication process, current devices designed for the brain typically interface with only hundreds of cells at a time and are unable to achieve cell type–specific integration with the tissue (*1*, *2*). An alternative approach to tackle this problem is by genetically programming specific cells within intact biological systems to build artificial structures with the desired form and function in situ. It had previously been shown that conductive polymers can be directly synthesized on cells in living tissue with electrochemical polymerization (*3*) or in organisms and tissues with native oxidative environments (*4*–*6*) or oxidative enzymes (*7*). However, none of these approaches enabled the critical goal of cellular specificity. We have taken the first step in this direction with the launch of the approach of genetically targeted chemical assembly (GTCA) (*8*), which uses cell-specific genetic information to guide neurons to initiate deposition of polymer materials in situ with a variety of electrical conduction properties. Specifically, an ascorbate peroxidase, Apex2 (*9*), was expressed in neurons as the catalyst and was used to initiate hydrogen peroxide (H_2_O_2_)–enabled oxidative polymerization of either conductive polymers or insulating polymers. Electrophysiological and behavioral analyses were used to confirm that the genetically targeted assembly of functional polymers remodeled membrane properties without requiring use of transgenic animal lines and successfully modulated cell type–specific behaviors in freely moving animals.

Despite this initial success, the procedures previously used for the proof-of-concept system displayed a major limitation: Apex2 was not specifically targeted to the extracellular side of the plasma membrane alone, and the majority of Apex2 was retained intracellularly (fig. S1). Developing a process that could efficiently place the reaction centers fully in the extracellular space, on the external side of the membrane, became critical to creating further applications of this biomanufacturing platform for the following reasons. First, living cells with intact membranes will not typically be permeable to large precursors or materials; thus, insufficient membrane display of enzymes may lead to low yield for chemical assembly in living systems. Second, increasing the number of enzymes catalyzing the reactions (leveraging extracellular space) may allow reduced concentrations of reagents important for setting reaction conditions (such as H_2_O_2_) and thereby improve biocompatibility. Third, localizing reactions to the extracellular space may limit adverse effects on native intracellular chemistry; previous reports have demonstrated that certain intracellular polymerization reactions may be toxic to cells and can induce apoptosis (*5*, *10*, *11*). Last, beyond membrane localization, many other avenues for advancing the technology also exist; for example, Apex2 is not optimized for applications on the cell surface. Another well-characterized peroxidase, horseradish peroxidase (HRP), catalyzes the same reactions as Apex2, with faster kinetics and greater resistance to H_2_O_2_-induced inactivation (*12*).

Here, we present a next-generation GTCA for targeted polymer assembly with HRP highly localized on the plasma membrane of primary neurons with minimal intracellular retention. Polymers synthesized extracellularly by this approach form dense clusters around the living neuronal membranes of interest, and neurons remained viable after acute polymerization. Last, this membrane localization method was shown to be readily adaptable for anchoring other proteins, enabling exploration of diverse alternative GTCA strategies.

## RESULTS

### In situ GTCA of polymers on the surface of neurons

**Fig. 1.**
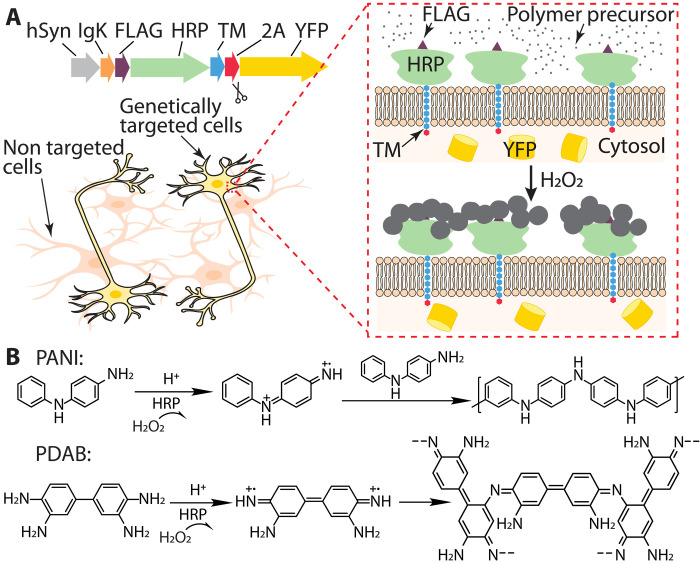
Schematics for next-generation GTCA in neurons. (**A**) Top, construct backbone for expressing membrane-displayed HRP. The plasmid contains the neuron-specific *hSyn* promoter, followed by sequences coding for the *IgK* leader, FLAG tags for antibody detection, HRP, a TM domain as the membrane targeting anchor, 2A self-cleaving peptides, and enhanced YFP. The targeted cells are expected to express membrane-displayed HRP and cytosolic YFP. Bottom, polymer assembly occurs specifically on the extracellular membrane of enzyme-targeted cells expressing cytosolic YFP (yellow). Expanded inset (red dashed line box) shows HRP anchored on the cell membrane enabling extracellular HRP/H_2_O_2_-catalyzed polymerization. Polymer precursors form dark-colored aggregates deposited on the cell surface. Cells without yellow cytoplasmic coloration represent nonenzyme-targeted cells. (**B**) HRP-mediated oxidative polymerization of PANI (top) and PDAB (bottom) from polymer precursors: *N*-phenyl-p-phenylenediamine (aniline dimer) and DAB, respectively.

Figure 1A depicts construct design for expressing a membrane-displayed form of the peroxidase HRP in primary neurons, using the neuron-specific human Synapsin (*hSyn*) promoter, followed by sequence encoding an *IgK* leader that initially directs the protein for membrane insertion in the endoplasmic reticulum (ER), FLAG tags for antibody detection, HRP itself, a transmembrane (TM) domain as the membrane anchor, 2A self-cleaving peptides, and enhanced yellow fluorescent protein (YFP). Targeted neurons thus are anticipated to express extracellular membrane–displayed HRP and cytosolic YFP, and HRP location can be determined by staining with antibodies targeting the FLAG tags. Upon addition of small-molecule polymer precursors and H_2_O_2_, the membrane-displayed HRPs act as reaction centers, facilitating oxidative radical polymerization on targeted neurons. Because of the low solubility of the resulting polymers, these synthesized polymers are expected to be not only extracellularly localized but also deposited onto the targeted cell membrane. Here, we initially selected two polymer precursors, *N*-phenyl-p-phenylenediamine (aniline dimer) and 3,3′-diaminobenzidine (DAB monomer), for the deposition of a conductive polymer, polyaniline (PANI), and a nonconductive polymer, poly(3,3′-diaminobenzidine) (PDAB), respectively (Fig. 1B). Both reactions occur in biocompatible aqueous solutions with 0.05 mM H_2_O_2_ unless otherwise specified.

### Membrane localization of HRP and evaluation of peroxidase activity

**Fig. 2.**
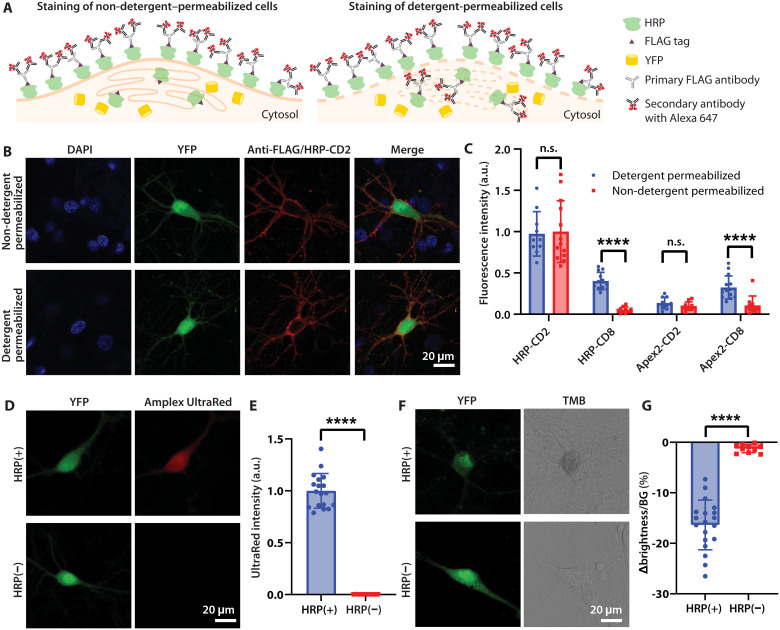
Membrane localization of peroxidases and evaluation of peroxidase activity. (**A**) Schematic: Quantitative evaluation of membrane expression. HRP fused to FLAG tag is detected with primary antibodies targeting FLAG and secondary antibodies coupled to Alexa Fluor 647. In non-detergent–permeabilized cells, only extracellular HRP can be detected. In detergent-permeabilized cells, both intracellular and extracellular HRP are labeled. (**B**) Confocal localization of HRP-CD2 in neurons. HRP localization in HRP(+) cells (here, expressing cytosolic YFP) was detected by immunostaining. Cell nuclei labeled with DAPI. (**C**) Fluorescence intensity comparison of antibody-stained detergent-permeabilized versus non-detergent–permeabilized neurons, expressing HRP or Apex2, with membrane anchors CD2 or CD8 (detergent-permeabilized cells: *N* = 12 cells for HRP-CD8, *N* = 14 cells for Apex2-CD8, and *N* = 10 cells for HRP-CD2 and Apex2-CD2; non-detergent–permeabilized cells: *N* = 13 cells for HRP-CD2 and *N* = 10 cells for HRP-CD8, Apex2-CD2, and Apex2-CD8). HRP-CD2, n.s. *P* = 0.8516, two-tailed unpaired *t* test; Apex2-CD2, n.s. *P* = 0.1829, two-tailed unpaired *t* test. Laser intensity and microscope settings were maintained consistent across conditions. (**D**) Evaluation of oxidative activity of HRP in living neurons expressing HRP-CD2 with the fluorogenic membrane-permeable dye Amplex UltraRed. The red fluorescent product of Amplex UltraRed oxidation indicates peroxidase activity. (**E**) Statistical comparison of UltraRed intensity; *N* = 18 cells for HRP(+), *N* = 10 cells for HRP(−). *y*-axis values were normalized to means of the HRP(+) condition. (**F**) Evaluation of oxidative activity of HRP in fixed neurons expressing HRP-CD2 with the chromogenic substrate TMB; blue reaction product (appearing dark in bright-field images) indicates peroxidase activity. (**G**) Statistical comparison of the ratio (expressed as %) of brightness difference between neuron and background (“∆brightness”) compared to background brightness (“BG”). *N* = 19 cells for HRP(+), *N* = 10 cells for HRP(−). Values shown: means ± SD; n.s., nonsignificant; ****P < 0.0001; two-tailed unpaired *t* test.

Native TM proteins are synthesized in the ER and transported to the Golgi apparatus and thereby reach the plasma membrane. Building upon the construct backbone described in Fig. 1, we sought to test a panel of native TM domains as membrane targeting anchors to most efficiently recruit native membrane trafficking machinery of primary neurons. We tested the TM domains of two T cell surface glycoproteins, *CD8*α (*13*) and *CD2* (*14*), for expressing either HRP or Apex2; to quantitatively compare membrane expression profiles resulting from these constructs, we performed antibody staining involving non-detergent–permeabilized or detergent-permeabilized cells (Fig. 2A). Specifically, HRP or Apex2 fused to FLAG tags were detected with primary antibodies targeting FLAG, which then could be labeled with secondary antibodies conjugated with Alexa Fluor 647 fluorophores. Under these conditions, in non-detergent–permeabilized cells, chiefly membrane-anchored enzymes could be selectively detected, whereas in detergent-permeabilized cells, both intracellular and extracellular enzymes could be robustly labeled.

Four days after transfection with plasmid DNA, enzyme localization in HRP(+) and Apex2(+) cells (i.e., here, cells expressing cytosolic YFP) was detected by immunostaining, and cell nuclei were labeled with 4′,6-diamidino-2-phenylindole (DAPI). Representative confocal microscopy of the HRP-CD2 neurons (Fig. 2B) revealed uniform membrane-associated antibody fluorescence on the soma and neurites in the anti-FLAG/Alexa Fluor 647 imaging channel. Similarity in HRP localization in detergent-permeabilized and non-detergent–permeabilized cells confirmed robust membrane-trafficking properties of CD2 under these conditions. We compared the fluorescence intensity of antibody staining in neurons expressing HRP-CD2, HRP-CD8, Apex2-CD2, and Apex2-CD8 (Fig. 2C); the percentage of enzyme-targeted immunofluorescence that was membrane displayed (relative to total expression levels) was quantified in these conditions as ~100, 13, 72, and 25%, respectively. CD2 thus exhibited more efficient membrane-anchoring properties than CD8 (fig. S2), and furthermore, the overall expression level of HRP-CD2 was higher than that of Apex2-CD2. Polymerization results confirmed that reactions on live HRP-CD2 neurons were much faster than on Apex2-CD2 neurons (fig. S3). We therefore used HRP-CD2 neurons as HRP(+) cells for the rest of the present work, with YFP-CD2 neurons as negative control HRP(−) cells.

Next, we tested HRP activity in live and fixed neurons using two peroxidase substrates: Amplex UltraRed and 3,3′,5,5′-tetramethylbenzidine (TMB), respectively. Amplex UltraRed is a fluorogenic substrate for HRP that produces bright red membrane-permeable product after oxidation (*15*); fluorescence images confirmed that HRP(+) but not HRP(−) neurons turned red after reaction (Fig. 2, D and E). TMB is a chromogenic substrate, which forms a water-soluble blue reaction product upon oxidation; bright-field images confirmed that the coloration change only occurred in HRP(+) neurons (Fig. 2, F and G).

### In situ polymer deposition and morphological characterizations

**Fig. 3.**
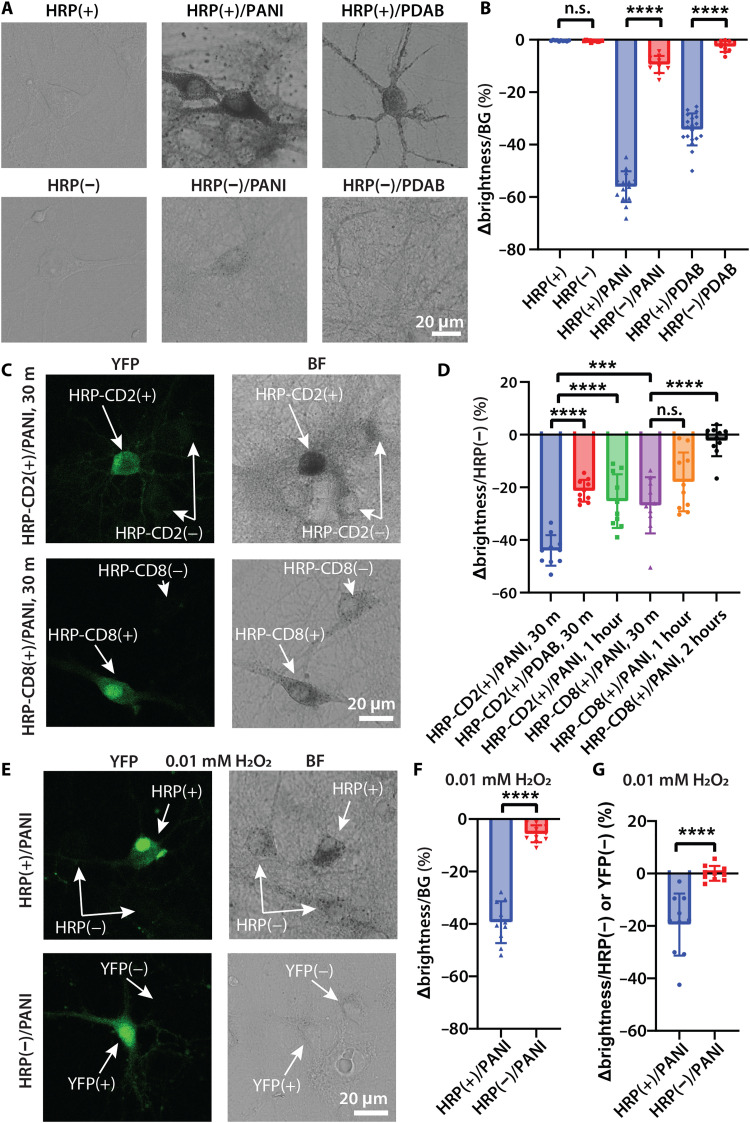
In situ genetically targeted polymer deposition on living neuronal membranes. (**A**) Bright-field images of live neurons with and without polymer deposition using the default reaction conditions for 30 min. (**B**) Statistical comparison of the ratio (expressed as %) of brightness difference between neuron and background (∆brightness) compared to background brightness (BG). *N* = 10 cells for HRP(+), HRP(−), HRP(−)/PANI, and HRP(−)/PDAB; *N* = 15 cells for HRP(+)/PANI; *N* = 19 cells for HRP(+)/PDAB. HRP(+) versus HRP(−), n.s., *P* = 0.1845, two-tailed unpaired *t* test. (**C**) Fluorescence and bright-field (BF) images of live HRP-CD2(+) and HRP-CD8(+) neurons showing transfected and nontransfected cells after PANI polymerization. (**D**) Statistical comparison of the ratio (expressed as %) of brightness difference between transfected HRP-CD2(+) and HRP-CD8(+) neurons and nontransfected neurons (∆brightness) compared to nontransfected neuron brightness [“HRP(−)”] after 30-min to 2-hour reaction times. *N* = 10 pairs of cells for each group. HRP-CD8(+)/PANI, 30 min versus HRP-CD8(+)/PANI, 1 hour, n.s. *P* = 0.0850, two-tailed unpaired *t* test. (**E**) Fluorescence and BF images of live HRP(+) and HRP(−) neurons after PANI polymerization using 0.01 mM H_2_O_2_ while using the same monomer concentration for 30 min. (**F**) Ratio of brightness difference between transfected neuron and background (∆brightness) in (E) compared to background brightness (BG). *N* = 10 cells for each group. (**G**) Ratio of brightness difference between transfected and nontransfected HRP(+) and YFP(+) neurons (∆brightness) compared to nontransfected neuron brightness [HRP(−) or YFP(−)]. *N* = 10 pairs of cells for each group. Values shown are means ± SD; n.s., nonsignificant; ***P < 0.001; ****P < 0.0001; two-tailed unpaired *t* test.

Next, we performed polymerization reactions for PANI and PDAB on HRP(+) and HRP(−) neurons. Bright-field images showed that HRP(+) neurons exhibited dark-colored reaction products, but HRP(−) neurons did not (Fig. 3A). Reaction progress was quantified as the decrease in brightness of cells relative to background (Fig. 3B). PANI formed densely distributed aggregates, while PDAB formed a thin uniform coating with scattered nonuniform aggregates. Selectivity of reactions could be conveniently assessed in transfected neurons (i.e., cells expressing strong cytosolic YFP signals) versus nontransfected neurons (i.e., cells with very low or without YFP signals) in the same field of view. When we performed PANI and PDAB polymerization on coverslips with live HRP-CD2(+) [i.e., HRP(+)] neurons, transfected cells became substantially darker than the neighboring nontransfected cells (Fig. 3C and fig. S4). After the same reaction on HRP-CD8(+) cells (Fig. 3C), statistical comparison of the brightness difference ratio (Fig. 3D) revealed significantly better selectivity in HRP-CD2(+) neurons versus HRP-CD8(+) neurons.

Notably, with longer reaction time, selectivity dropped for both HRP-CD2(+) and HRP-CD8(+) neurons (Fig. 3D and fig. S4), indicating that the reactions became dominated by the background polymerization. In addition, although 0.05 mM H_2_O_2_ has been proven to be biocompatible (*8*), considering our substantially improved membrane localization, we explored further reducing the H_2_O_2_ concentration to 0.01 mM while using the same monomer concentration and the same reaction time (Fig. 3, E to G). Again, bright-field images of live HRP(+) and HRP(−) neurons showed dark-colored aggregates selectively deposited on transfected HRP(+) cells. We found that it was possible to further decrease the H_2_O_2_ concentration to 0.002 mM, although selectivity was impaired (fig. S5). These results demonstrate the benefit of selective membrane functionalization to further improve biocompatibility of GTCA.

**Fig. 4.**
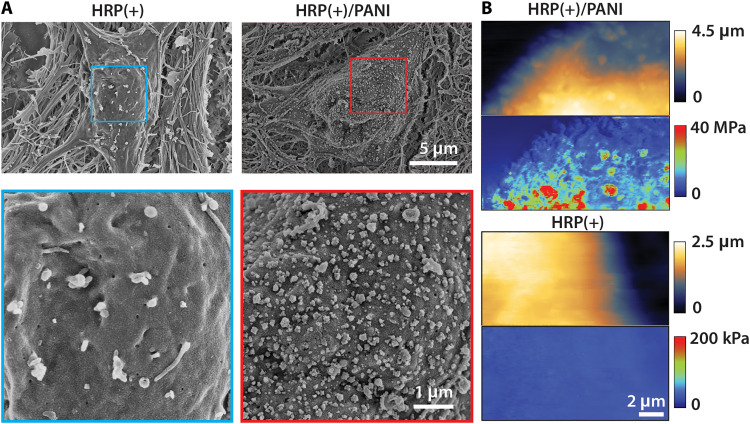
Morphological and mechanical characterizations of in situ deposited polymers. (**A**) Representative SEM images of neurons with and without 30 min polymer deposition. HRP(+)/PANI conditions form dense polymer aggregates on the membrane. Additional SEM images are included in fig. S6. (**B**) Representative AFM images of HRP(+) neurons with and without 30 min PANI deposition. Top, height channel; bottom, modulus map. Polymer particles exhibited substantially higher modulus compared to the membrane. Original AFM images are shown in fig. S8.

Scanning electron microscopy (SEM) imaging confirmed the morphologies of both polymer depositions (Fig. 4A and figs. S6 and S7). HRP(+)/PANI neurons formed dense clusters around the neuronal soma and neurites, with an average particle size of 120 ± 13 nm (*N* = 50 particles). By contrast, HRP(+) cells not exposed to PANI exhibited smooth membrane surfaces. The HRP(+)/PDAB neurons formed a thin layer with nonuniform aggregates of clusters, with an average particle size of 115 ± 11 nm (*N* = 50 particles) (fig. S7). Further comparison with the SEM images of HRP(−) cells confirmed localization specificity of this genetic targeting approach (fig. S7). We also used atomic force microscopy (AFM) to characterize the height and Derjaguin-Muller-Toporov modulus mappings of PANI particles on the neuronal membrane (Fig. 4B; see fig. S8 for original images). Height maps of HRP(+)/PANI cells again showed clear distribution of ~100 nm polymer particles, similar to those seen in SEM images and uniformly deposited across the membrane. A significant increase was observed in the modulus maps after polymerization, with polymer particles showing modulus values at least three orders of magnitude higher than on cells without polymerization (fig. S8). Notably, the membrane regions between particles also showed much higher modulus than the unmodified membrane, suggesting the presence of a thin layer of PANI uniformly coated on cells between the particles. Next-generation AFM measurements in the future might be suitable to further characterize this polymer layer and study how the membrane modulus increase changes cellular properties.

### Spectroscopic characterizations of in situ deposited polymers

**Fig. 5.**
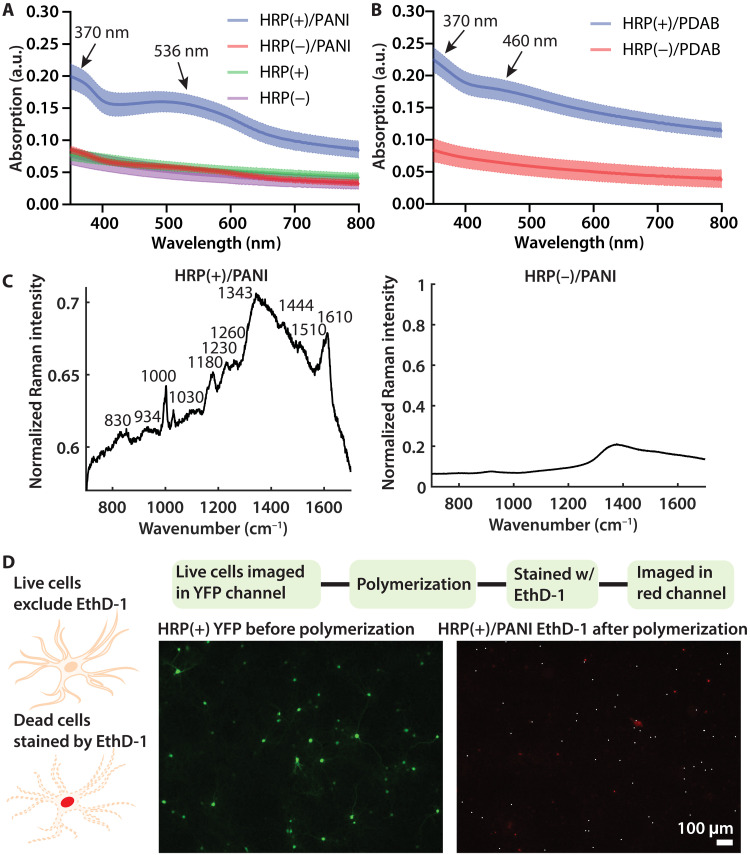
Spectroscopic characterizations of in situ deposited polymers and cell viability assay. (**A** and **B**) Normalized UV-Vis spectra of HRP(+) and HRP(−) neurons with and without PANI and PDAB deposition from *N* = 5 coverslips. Values shown: means ± SD. Arrows indicate absorption peaks. a.u., arbitrary units. (**C**) Raman spectroscopy analysis of the HRP(+) and HRP(−) after PANI deposition. Additional Raman results are included in figs. S9 and S10. (**D**) Cell viability test of polymerization conditions. Live HRP(+) cells were imaged with YFP and, after PANI deposition for 30 min, stained with EthD-1, a red fluorophore that selectively stains dead cells with damaged membranes. White dots in the EthD-1 image mark locations of the HRP(+) cells. Lack of white dot overlay with red cells reveals that HRP(+) neurons remain viable after polymerization. Additional cell viability tests and positive controls are included in figs. S11 and S12.

To characterize the chemical composition of in situ deposited polymers, we used ultraviolet-visible (UV-Vis) absorption spectroscopy to compare with spectra previously reported for PANI and PDAB (*N* = 5 coverslips for each condition) (Fig. 5, A and B). HRP(+)/PANI spectra exhibit an absorption peak at approximately 370 nm that is attributable to π–π* transition of the benzenoid ring. The peak at 536 nm corresponds to intramolecular transition of benzenoid rings into quinoid rings (*16*, *17*). The HRP(+)/PDAB spectra also exhibit an absorption peak at 370 nm, and the 460 nm peak corresponds to the characteristic absorption of PDAB (*18*). Last, confocal Raman microscopy was used to examine single cells with and without PANI (Fig. 5C and fig. S9) and PDAB (fig. S10). The bands at 830, 1000, and 1030 cm^−1^ correspond to the in-plane deformation of the rings. The bands at 1230, 1260, 1343, and 1444 cm^−1^ are attributable to the C─N and C═N stretching vibrations. The 1180 and 1510 cm^−1^ are from deformation vibrations of C─H and N─H, and the 934 and 1610 cm^−1^ correspond to the stretching vibration of C─C (*19*). Together, these results confirmed that PANI was synthesized under these conditions (Fig. 5C).

### Cell viability determination

We used a cell viability assay to monitor HRP(+) cells before and after polymerization (Fig. 5D and figs. S8 and S9). YFP signals of live HRP(+) cells were assessed, and after PANI and PDAB deposition, cells were stained with ethidium homodimer (EthD-1, a red fluorophore that only stains nucleic acids in dead cells). The locations of HRP(+) cells are marked in the EthD-1 channel with white dots (Fig. 5D); lack of overlay with red cells reveals that HRP(+) neurons remain viable after polymerization. In comparison, the positive control for EthD-1 staining on fixed cells confirmed that dead cells were specifically labeled with proper sensitivity using the same procedure (fig. S11); this cell viability test was repeated on *N* = 5 coverslips for each polymerization condition (fig. S12).

### Exploring alternative GTCA strategies with other membrane-anchored proteins

Beyond peroxidase-controlled oxidative polymerization, many alternative strategies for advancing GTCA could be explored, powered by this robust extracellular membrane–anchoring CD2 strategy. We next tested, and successfully demonstrated, two complementary GTCA strategies for cell-specific synthesis.

**Fig. 6.**
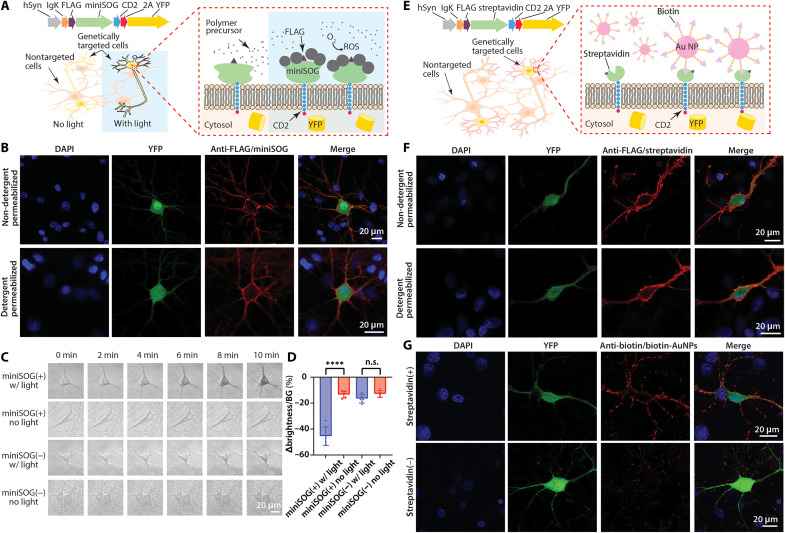
Exploring alternative GTCA strategies. (**A**) Genetically encoded photosensitizer enabled light-controlled polymerization on the surface of neurons. Diagram: Upon blue light illumination, the miniSOG photosensitizer expressed on genetically targeted cells may convert oxygen to ROS, thereby catalyzing the oxidative polymerization reaction. (**B**) Confocal microscopy of expression of miniSOG in neurons 7 days after transfection, using the same approach as Fig. 2B. The similarity in miniSOG expression and localization in permeabilized and nonpermeabilized cells indicates robust membrane localization. (**C**) Bright-field images of live miniSOG(+) and miniSOG(−) neurons with PANI deposition under 20 mW/mm^2^ blue light illumination. (**D**) Statistical comparison of the ratio (expressed as %) of brightness difference between neuron and background (∆brightness) compared to background brightness (BG) after 10 min polymerization. *N* = 5 cells. Values are means ± SD; n.s., nonsignificant, ****P < 0.0001; two-tailed, unpaired, *t* test. (**E**) Genetically encoded conjugation of presynthesized materials on the surface of neurons. Diagram: Cells expressing membrane-anchored streptavidin may selectively bind biotin-conjugated materials, such as Au NPs. (**F**) Confocal microscopy of expression and membrane localization of streptavidin in neurons 4 days after transfection. (**G**) Confocal microscopy of biotin–Au NP binding on streptavidin(+) and streptavidin(−) cells after incubating live neurons in Au NP solutions. Cells were then assayed for biotin localization. Additional staining results showing Au NP binding are included in fig. S13.

First, we tested genetically targeted light-controlled oxidative polymerization (Fig. 6, A to D). In HRP-catalyzed polymerization reactions, all cells are immersed in polymer precursor solutions with little opportunity for spatial resolution beyond genetic specificity; moreover, the HRP/H_2_O_2_ system can only trigger a one-time oxidative reaction, rather than allowing patterning over time. To better approach the complexity and plasticity of biological structures, we explored a light-controlled approach, using a genetically encoded photosensitizer [miniSOG (*20*)] that produces reactive oxygen species upon illumination and could thereby create a membrane-localized reaction center for photopolymerization (Fig. 6A); we and others in 2022 had demonstrated an initial version of this possibility but not yet with the next-generation GTCA targeting strategy shown here (*21*, *22*). We stained cells 7 days after transfection using our non-detergent–permeabilized and detergent-permeabilized protocols; representative confocal imaging of miniSOG-CD2 neurons (Fig. 6B) showed efficient membrane-anchoring of miniSOG. Next, we performed polymerization of PANI on miniSOG(+) and miniSOG(−) neurons with or without illumination with blue light for 10 min (Fig. 6C). Bright-field images showed that neurons exhibited dark polymer deposits as the reaction progressed, and analysis revealed significantly increased polymerization reaction progression on miniSOG(+) neurons under illumination (Fig. 6D).

Second, we tested genetically targeted conjugation of presynthesized materials (Fig. 6, E to G). In our initial work on GTCA, we had focused on in situ assembly of functional materials on, and building from, the cell membrane. Many types of reactions fit in this regime of building reaction product from the cell—that is, de cellula—but only a subset of such reactions are biocompatible for live cells. Thus, we next explored anchoring partially synthesized materials to the cell—an ad cellula approach—by expressing the biotin-binding protein streptavidin (*23*) on neuronal membranes and linking biotin to presynthesized materials, in this case, gold nanoparticles (Au NPs) (Fig. 6E); in this way, selective localization of materials to cells with streptavidin on the surface might then be achieved. Four days after transfection, confocal imaging showed uniform staining of streptavidin on the neuronal membranes (Fig. 6F). After incubating the live streptavidin(+) and streptavidin(−) cells in biotin-conjugated Au NP solutions, we found uniform and selective membrane binding of Au NPs on the somata and neurites of streptavidin(+) cells (Fig. 6G and fig. S13).

## DISCUSSION

Here, we design and deploy a method for genetically specifying cells to synthesize functional materials that allows precise and robust targeting of critical enzymes to the extracellular space, while the enzymes (and reaction product) remain localized to the targeted plasma membrane, thereby addressing a key limitation of the GTCA platform. We found that polymers synthesized in situ on primary neurons selectively formed dense clusters on the extracellular face of the surface plasma membrane, and we determined that neurons retained viability after the synthetic reaction was completed in this way. We also demonstrated that localizing reactions to extracellular space, beyond supporting biocompatibility, could also improve reaction yield and allow reduced concentrations of reactants.

We anticipate that in the future, this approach may show generalized utility across a variety of application domains. It has already been shown that using GTCA to target deposition of conductive or insulating polymers on living neuronal membranes, to increase or decrease capacitance respectively, can modulate excitability (*8*). The improved targeting and efficiency of the approach shown here may support the formation in living animals of more complex structures and the modulation of more complex behaviors.

We here note that dense construction of conductive polymers, along with nonconductive polymer insulation, could be used to create conductive pathways between arbitrarily defined neurons, microcircuits, or nervous system regions, thereby effectively writing connections into living brains. Conductivity could be tuned by including different precursors, changing reactant concentrations and reaction time, and introducing doping agents. For such chronic in vivo applications wherein precursor solutions are initially injected into brain tissue, systematic biocompatibility tests should be performed, including measures of potential inflammatory responses to reactants and particles at different concentrations and different times after reaction [as we have shown previously (*8*)]. Notably, ~100 nm NPs injected into the brain can exhibit clearance half-lives of several days (*24*)—likely through a microglia-mediated paravascular glymphatic pathway (*25*)—although the transportation kinetics and mechanism that would clear in situ synthesized polymer deposits are still unknown.

The flexibility and modularity of this single-vector approach may be adapted not only to localize a broad diversity of proteins to primary neuronal membranes (as with miniSOG and streptavidin as shown here) but also to specify targeting via the rapidly expanding panel of promoters and enhancers available for targeting specific cell types in the brain and beyond. For example, astrocytes could be targeted with GTCA to express HRP or other proteins by exchanging the neuron-specific *hSyn* promoter for the astrocyte-specific *GFAP* promoter (*26*); among other applications, making glial cells conductive in this way could be used to promote stability of chronic neural recording/stimulation even with conventional electrodes, which otherwise are known to promote reactive formation of glial scar tissue that insulates neurons from the electrodes (*27*, *28*). This platform may be readily extended to a range of cell types, reactants (such as other redox-sensitive molecules), catalysts (such as enzymes or modulators), or reaction conditions (through modulating pH, light, chemical, redox, and electrical signals) (*29*), thereby enabling investigators to establish diverse classes of specific and seamless integration with living biological systems beyond conventional design and assembly capabilities alone.

## MATERIALS AND METHODS

### Plasmid constructs

The following constructs were designed in SnapGene 5.1.7 and cloned into adeno-associated virus (AAV) plasmids with the human Synapsin (*hSyn*) promoter. All sequences were confirmed with Sanger sequencing (Azenta).

HRP-CD2 or HRP(+): hSyn-IgK-FLAGx3-HRP-CD2-p2A-t2A-YFP

HRP-CD8: hSyn-IgK-FLAGx3-HRP-CD8-p2A-t2A-YFP

Apex2-CD2: hSyn-IgK-FLAGx3-Apex2-CD2-p2A-t2A-YFP

Apex2-CD8: hSyn-IgK-FLAGx3-Apex2-CD8-p2A-t2A-YFP

YFP-CD2 or HRP(−) or miniSOG(−) or streptavidin(−): hSyn-IgK-FLAGx3-YFP-CD2-p2A-t2A-YFP

miniSOG-CD2 or miniSOG(+): hSyn-IgK-FLAGx3-miniSOG-CD2-p2A-t2A-YFP

streptavidin-CD2 or streptavidin(+): hSyn-IgK-FLAGx3-streptavidin-CD2-p2A-t2A-YFP

### Neuron culture, transfection, and antibody staining

Primary cultures of postnatal hippocampal rat neurons were prepared on 12 mm coverslips in 24-well plates, as described previously (*8*). Cells were transfected 6 to 7 days in vitro with various constructs. For each coverslip to be transfected, a DNA-CaCl_2_ mix containing the following reagents was prepared using a calcium phosphate transfection kit (Invitrogen, 44-0052): 1 μg of plasmid DNA, 1 μg of salmon sperm DNA (Invitrogen, 15632-011), 1.875 μl of 2 M CaCl_2_, and sterile water were added for a total volume of 15 μl. Last, 15 μl of 2× Hepes-buffered saline (HBS) was added, and the resulting 30 μl mix was incubated at room temperature for 20 min. The growth medium from each well was removed, saved, and replaced with 400 μl of prewarmed minimal essential medium (MEM, Invitrogen, 11095114); the DNA-CaCl_2_-HBS mix was added dropwise into each well, and the plates were returned to the culture incubator for 60 min. Each coverslip was then washed three times with 1 ml of prewarmed MEM and placed back to the original neuronal growth medium. Neurons were used 4 to 6 days after transfection. To characterize membrane localization of HRP, Apex2, and streptavidin 4 days after transfection, cultured neurons expressing different constructs were stained using two protocols and imaged with a Leica TCS SP8 confocal laser scanning microscope. Membrane localization of miniSOG cells and light-controlled polymerization were performed 7 days after transfection.

#### Non-detergent–permeabilized staining

Cultured neurons were washed three times with 1 ml of prewarmed serum-free Neurobasal medium (Gibco, 21103049) supplemented with 4% B-27 (Gibco, 17504044) and 2 mM Glutamax (Gibco, 35050061), fixed in 4% paraformaldehyde (PFA) at room temperature for 15 min, and washed three times with phosphate-buffered saline (PBS). Cells were blocked with PBS containing 5% normal goat serum (Jackson ImmunoResearch, 005-000-121) at room temperature for 30 min and then stained with primary antibody against FLAG (DDDDK tag, Abcam, ab1162) at 1:200 dilution with 5% goat serum at 37°C for 1 hour and washed three times with PBS. Cells were then stained with Alexa Fluor 647 goat anti-rabbit secondary antibody (Abcam, ab150087) at 1:500 dilution with 5% goat serum at 37°C for 1 hour and washed three times with PBS. Cells were finally permeabilized in PBS containing 5% goat serum and 0.03% Triton X-100 at room temperature for 10 min, and mounted on slides using VECTASHIELD hardSet antifade mounting medium with DAPI (Vector Laboratories).

#### Detergent-permeabilized staining

Cultured neurons were washed and fixed in 4% PFA, as described above. Cells were then blocked and permeabilized with PBS containing 5% goat serum and 0.03% Triton X-100 at room temperature for 1 hour and then stained with primary antibody and secondary antibody and mounted with DAPI, as described above.

### Evaluation of peroxidase activity

#### Amplex UltraRed staining

Live neurons in media were placed on ice for 3 min, incubated in 200 μl of 50 μM Amplex UltraRed (Invitrogen, A36006) in ice-cold Tyrode’s solution (125 mM NaCl, 2 mM KCl, 2 mM MgCl_2_, 2 mM CaCl_2_, 30 mM glucose, and 25 mM Hepes, titrated to pH 7.35 with NaOH and adjusted osmolarity to 298) containing 0.05 mM H_2_O_2_ for 5 min, washed with 1 ml of fresh ice-cold Tyrode’s solution, and imaged with a Leica SP8 confocal microscope within 10 min.

#### TMB staining

Cultured neurons were washed three times in media, fixed in 4% PFA at room temperature for 15 min, and washed three times with PBS. TMB solution was prepared by diluting the liquid TMB substrate containing 1.46 mM TMB and 2.21 mM H_2_O_2_ (Calbiochem, 3905420) 30 times in PBS. Fixed neurons were placed in the TMB solution for 10 min, and imaged with Leica SP8 confocal microscope within 10 min.

### Polymerization reactions

For polymerization reactions, cells were incubated in a mixture of polymer precursor solutions and hydrogen peroxide solution for 30 min unless specified and washed three times in Tyrode’s solution. All solutions were prepared fresh each time. Aniline dimer solution (3 mM) was prepared by dissolving 5.5 mg of *N*-phenyl-p-phenylenediamine (Sigma-Aldrich, 241393) in 10 ml of Tyrode’s solution ~20 hours at room temperature using a magnetic stir bar. DAB solution (10 mM) was prepared by dissolving 36 mg of 3,3′-diaminobenzidine tetrahydrochloride hydrate (Sigma-Aldrich, D5637) in 10 ml of Tyrode’s solution. The pH of the solution was adjusted to 7.35 by 1 M NaOH solution. When ready to perform the reactions, the above solutions were filtered with 0.45 μm syringe filters (Thermo Fisher Scientific), and H_2_O_2_ (EMD Millipore, 386790) was added to the solutions for a final concentration of 0.05 mM unless specified.

### Light-controlled polymerization reaction

Live miniSOG(+) or miniSOG(−) neurons on coverslips were immersed in the 3 mM aniline dimer solution and placed on a Leica CTR6000 inverted microscope equipped with a Lumencor LED Fluorescence Illuminator. The coverslips were illuminated with blue light passed through an excitation filter 434/17 filter and a 40× objective at 20 mW/mm^2^ intensity. Bright-field images were taken after every minute of illumination over 10 min.

### Biotin-conjugated Au NPs binding and imaging

Culture medium containing Au NPs was prepared by mixing 5 μl of 100 nm biotin-polyethylene glycol–conjugated Au NP suspension (Cytodiagnostics Inc., CGB5K-100-25) into 1 ml of neuron culture media (final concentration of NPs ~32 fM). Live streptavidin(+) and streptavidin(−) neurons on coverslips were incubated in culture medium containing Au NPs for 1 hour at 37°C in the incubator. The cells were then washed thoroughly five times in culture medium, fixed in 4% PFA for 15 min at room temperature, and stained using the detergent-permeabilized staining protocol, with primary antibody against biotin (Abcam, ab53494) at 1:200 dilution, followed by Alexa Fluor 647 goat anti-rabbit secondary antibody, as described above.

### Scanning electron microscopy

Neurons were fixed with EM fix [2% glutaraldehyde (Electron Microscopy Sciences, 16020) with 4% PFA in 0.1 M Na Cacodylate buffer (Electron Microscopy Sciences, 11652)] at room temperature for 1 hour and then washed three times in PBS. After reactions, cells were returned to EM fix and stored at 4°C for 1 hour and then washed three times with 0.1 M Na Cacodylate buffer for 10 min each time. The buffer was then replaced with 1% aqueous osmium tetroxide (Electron Microscopy Sciences, 19192), at room temperature for 1 hour and washed with deionized (DI) water twice for 10 min each time. Samples were then dehydrated in a series of ethanol washes (50, 70, 95, 100, and 100%) for 10 min each and then incubated in a series of hexamethyldisilazane (HMDS):ethanol mixtures (1:9, 2:8, 3:7, 4:6, 5:5, 6:4, 7:3, 8:2, 9:1) for 5 min each, followed by 100% HMDS twice for 20 min each time. The HMDS was then removed, and the coverslips were dried overnight in a chemical hood. The samples were mounted with double-sided carbon tapes (Ted Pella, 16085-1) on aluminum tabs (Ted Pella, 16084-1), coated with 4 nm gold with a Leica EM ACE600 sputter coater, and imaged with a Zeiss Sigma SEM.

### Atomic force microscopy

Morphology and modulus mappings were collected using a Bruker BioScope Resolve BioAFM with a SCANASYST-FLUID+ probe (Bruker) with a nominal spring constant of 0.7 N/m and a tip radius of 2 nm on pre- and post-reacted fixed neurons in Tyrode’s solution at room temperature. Nanomechanical maps were recorded in the Peak Force Quantitative Nanomechanics (PF-QNM) mode while scanning across the cell membrane for the height images. All force modulation measurements were performed at a set point of ~1.2 nN with a PF frequency of 0.5 kHz and an amplitude of 300 nm. The scan resolution was set to 256 × 256 pixel with a scan rate of 0.5 Hz. The data were evaluated and depicted with Gwyddion SPM software. The histograms of the modulus channel were generated via a one-dimensional statistical function embedded in Gwyddion.

### UV-Vis spectrophotometry and Raman spectroscopy

Post-reacted, fixed neurons on glass coverslips were washed three times in PBS and three times in DI water, air dried, and mounted onto the sample holders. UV-Vis spectrophotometry was performed with Agilent Cary 6000i spectrophotometer. Raman spectroscopy was performed with Horiba XploRA confocal Raman microscope with a 785-nm laser using a 100× Olympus MPLan N objective.

### Cell viability assay

To examine cell viability of neurons after reaction, coverslips with live HRP(+) neurons were fixed on the sample platform of a Leica DMi8 microscope and imaged with the YFP signal. After polymerization (or fixation in 4% PFA at room temperature for 15 min and permeabilization with 0.03% Triton X-100 at room temperature for 15 min as the positive control), dead cells were stained red with 4 μM EthD-1 from Invitrogen LIVE/DEAD viability/cytotoxicity kit (Invitrogen, L3224) in Tyrode’s solution at room temperature for 30 min and imaged in the red channel.

### Data analysis

ImageJ software was used for imaging analysis, including fluorescence intensity and brightness measurements. For cell viability tests, the yellow and red channel images were concatenated, and the locations of cells expressing YFP were marked with white dots in the red channel. Spectroscopic results were plotted using Matlab R2022b. Statistical analyses for all data were performed with two-tailed unpaired *t* test using GraphPad Prism 9. Chemical structures were prepared using ChemDraw 21.0.
