# Supplementary material for: Genetically targeted chemical assembly of polymers specifically localized extracellularly to surface membranes of living neurons
Source: Sci Adv. 2023 Aug 9;9(32):eadi1870. doi: 10.1126/sciadv.adi1870 (PMC10411876; doi:10.1126/sciadv.adi1870)
Supplement: Supplementary file 1 — Figs. S1 to S13 [file sciadv.adi1870_sm.pdf]

Science Advances
AAAS

Supplementary Materials for

Genetically targeted chemical assembly of polymers specifically localized extracellularly to surface membranes of living neurons

Anqi Zhang *et al.*

Corresponding author: Karl Deisseroth, deissero@stanford.edu; Zhenan Bao, zbao@stanford.edu

Sci. Adv. **9**, eadi1870 (2023)
DOI: 10.1126/sciadv.adi1870

This PDF file includes:

Figs. S1 to S13

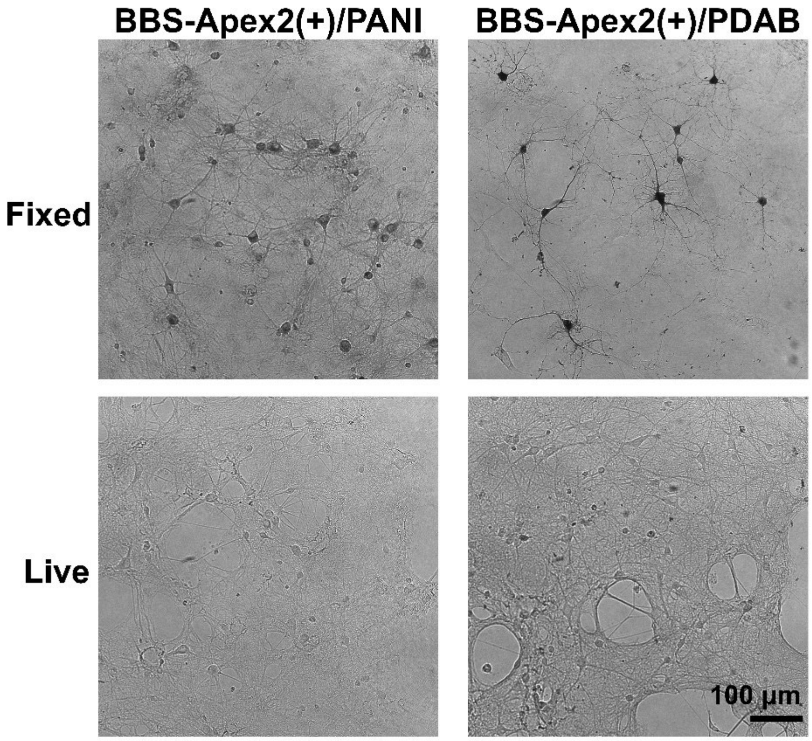

Fig. S1.

Persistence of significant APEX2 in the intracellular compartment when using the previous BBS motif method. The panels show comparison of polymerization on fixed vs. living neurons transfected with AAVdj virus encoding hSyn-BBS-Apex2-YFP using reaction conditions as described in Liu et al., Science 367, 1372-1376 (2020). Assembled polymers form dark aggregates in cells. In fixed neurons, where cell membranes are permeabilized during polymerization, both intracellular and extracellular Apex2 catalyzed polymerization. In living neurons, where cell membranes remain intact during polymerization, extracellular Apex2 more robustly catalyzed polymerization; fixed BBS-Apex2 neurons were darker than living neurons, consistent with the interpretation that Apex2 in previous work was still present intracellularly to a substantial extent.

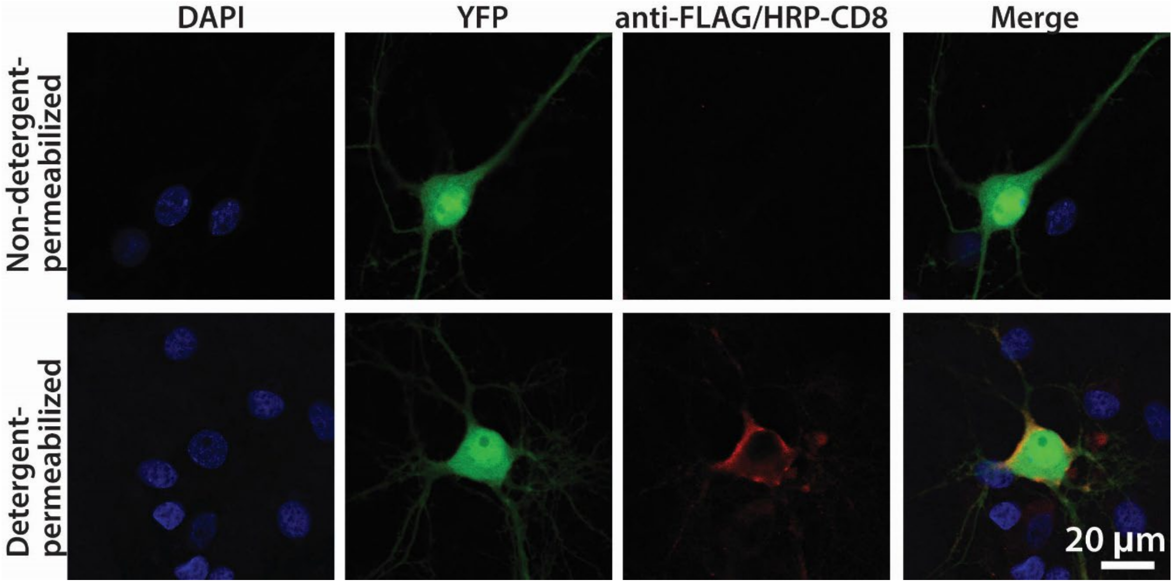

Fig. S2.

Confocal microscopy of expression and membrane localization of HRP-CD8 in neurons. Cells were stained using the same protocols as those with HRP-CD2 in Fig. 2B. The difference in HRP localization in detergent-permeabilized and non-detergent-permeabilized cells demonstrates that CD8 is not as efficient as CD2 for membrane-trafficking. Laser intensity and microscope settings were maintained to be consistent across conditions.

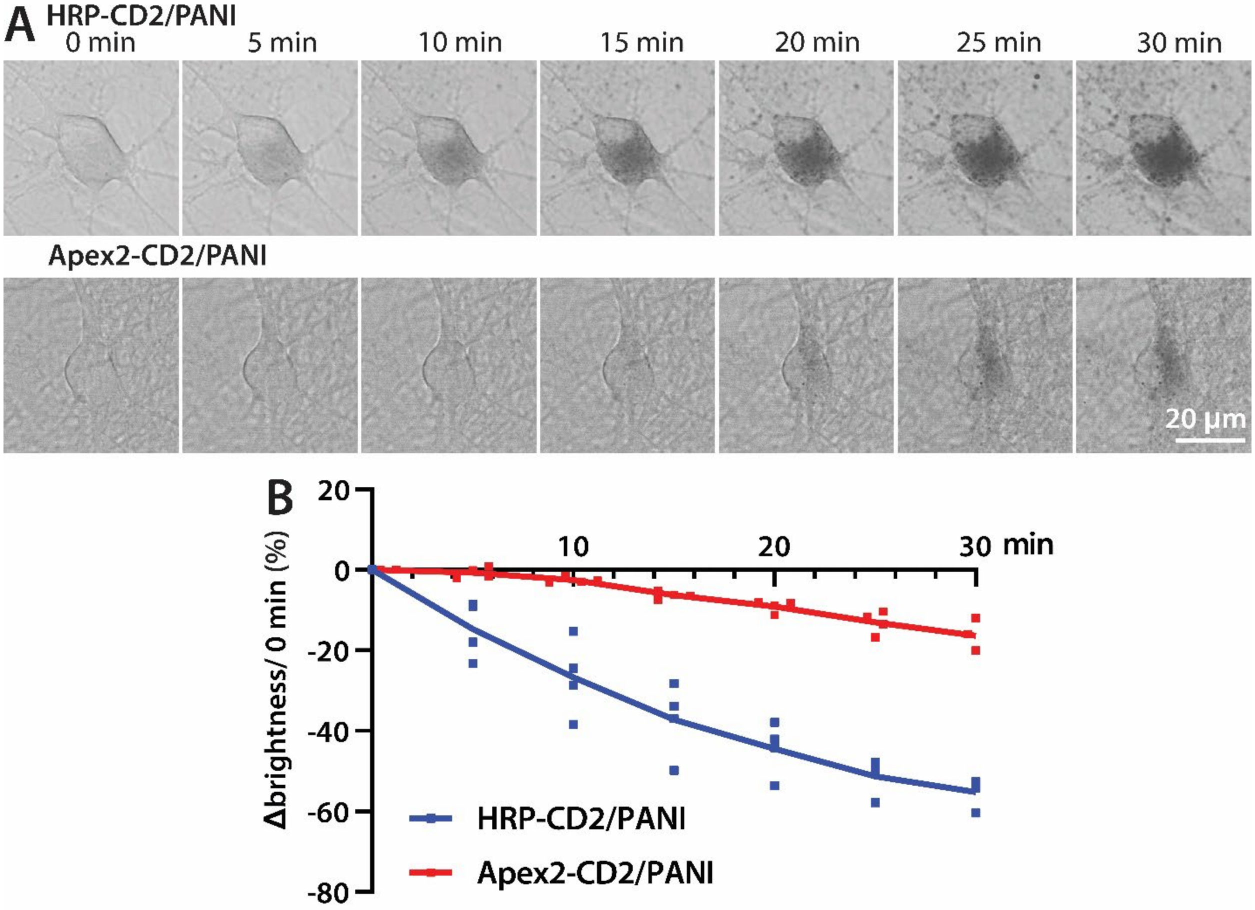

Fig. S3.

Comparison of PANI polymerization reactions on cells expressing HRP-CD2 and Apex2-CD2. (**A**) Bright field images of living neurons with PANI deposition over 30 min. (**B**) Comparison of the ratio (expressed as %) of brightness difference between neurons at each time point and before polymerization ("Δbrightness"), compared to before polymerization ("0 min"). N = 4 coverslips. Values are means. The polymerization rate on HRP-CD2 cells is faster than on Apex2-CD2 cells.

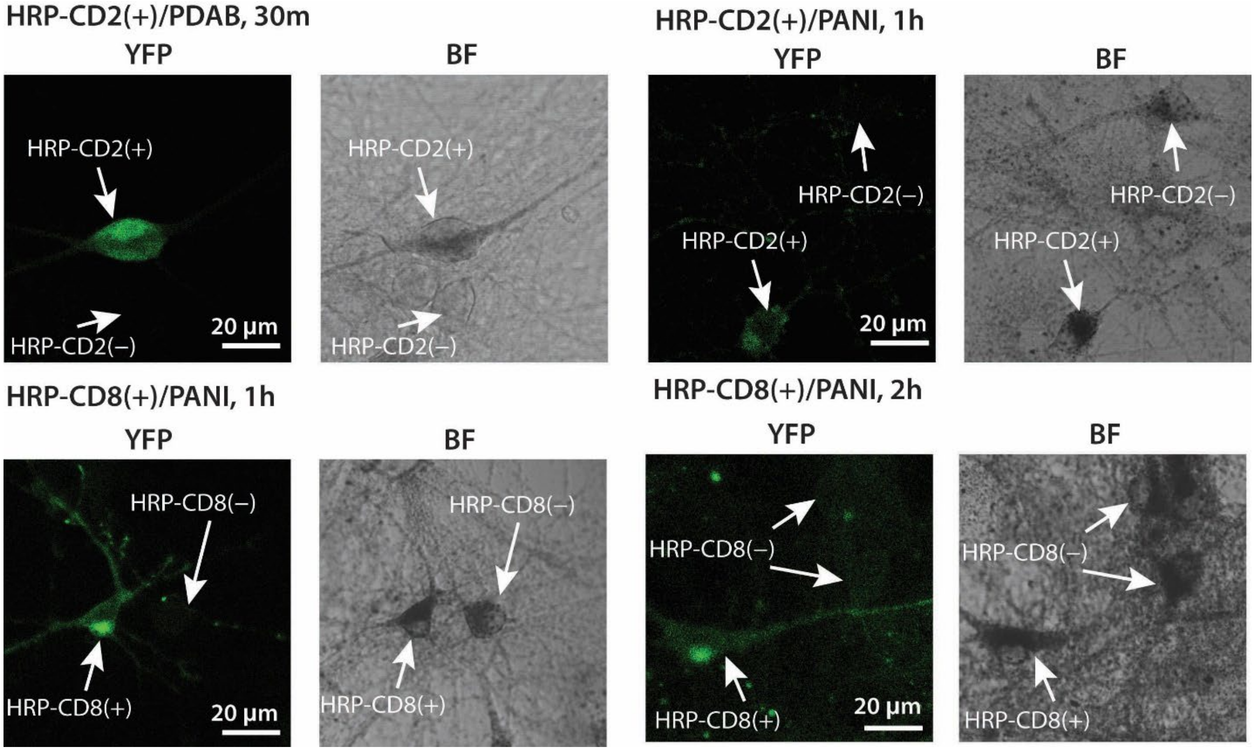

Fig. S4.

Reaction selectivity under different conditions. Fluorescence and bright field (BF) images of live HRP-CD2(+) (top) and HRP-CD8(+) (bottom) neurons showing transfected and non-transfected cells after PDAB polymerization for 30 min and PANI polymerization for 1 h, and PANI polymerization for 1 h and 2 h, respectively. Notably, after 2 h polymerization with HRP-CD2(+) cells, the reaction had progressed to such an extent that cells became extremely dark with reaction product and began to detach from the coverslip.

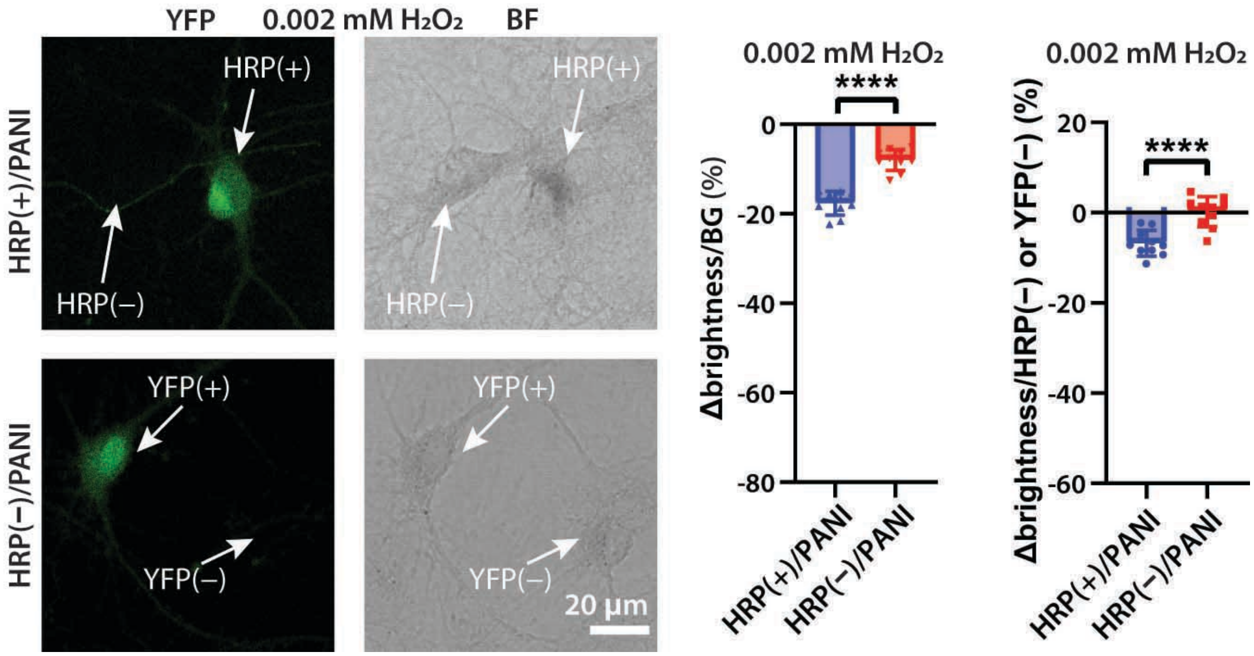

Fig. S5.

Polymerization with H_2O_2 concentration of 0.002 mM. Left, fluorescence and bright field (BF) images of live HRP(+) and HRP(−) neurons after PANI polymerization using 0.002 mM H_2O_2 while using the same monomer concentration for 30 min. Middle, ratio of brightness difference between transfected neuron and background ("Δbrightness"), compared to background brightness ("BG"). N = 10 cells for each group. Right, ratio of brightness difference between transfected and non-transfected HRP(+) and YFP(+) neurons ("Δbrightness"), compared to non-transfected neuron brightness ("HRP(−) or YFP(−)"). N = 10 pairs of cells for each group. Values shown are means ± s.d.; *****P* < 0.0001; two-tailed unpaired *t*-test.

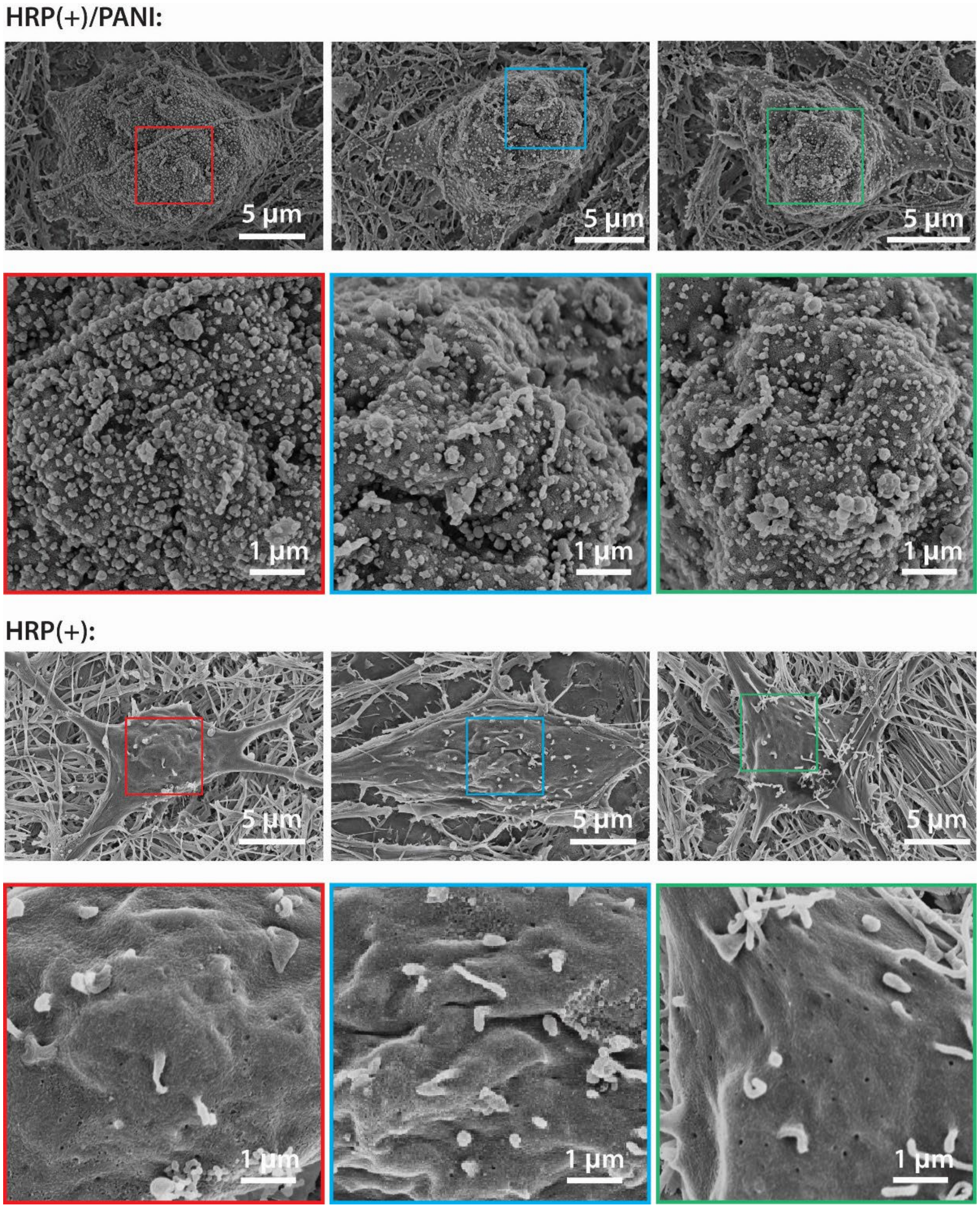

Fig. S6.

Additional SEM images of HRP(+)/PANI and HRP(+) neurons. These images complement Fig. 4A.

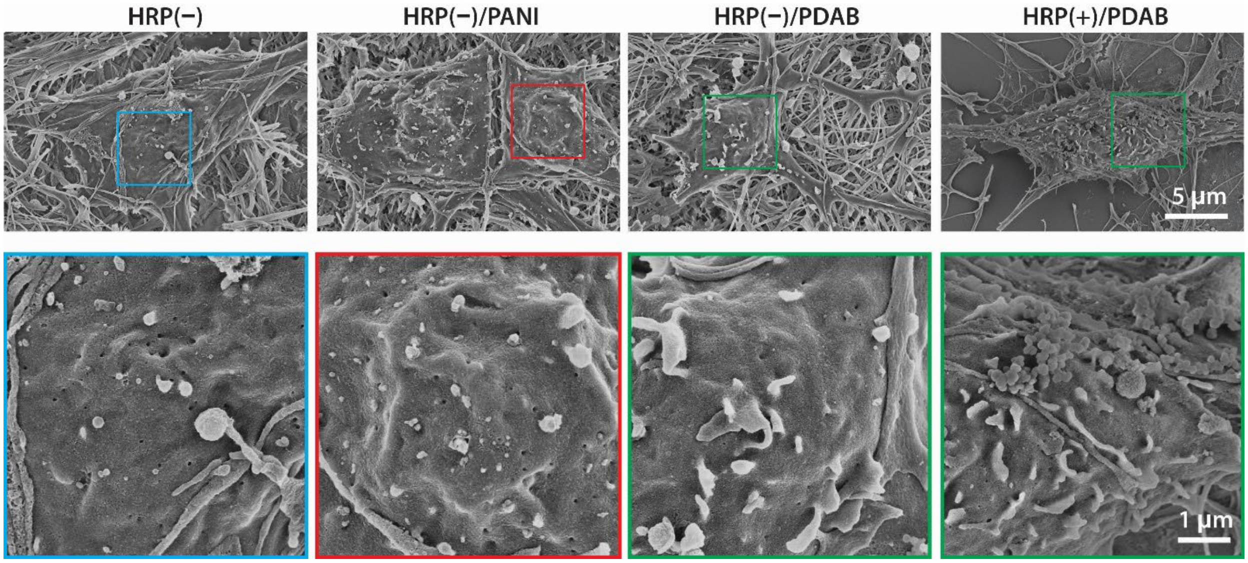

Fig. S7.

Additional SEM images of HRP(−) and HRP(+)/PDAB neurons. SEM images of HRP(−) neurons with and without PANI reaction, and of HRP(−) and HRP(+) neurons with PDAB reaction.

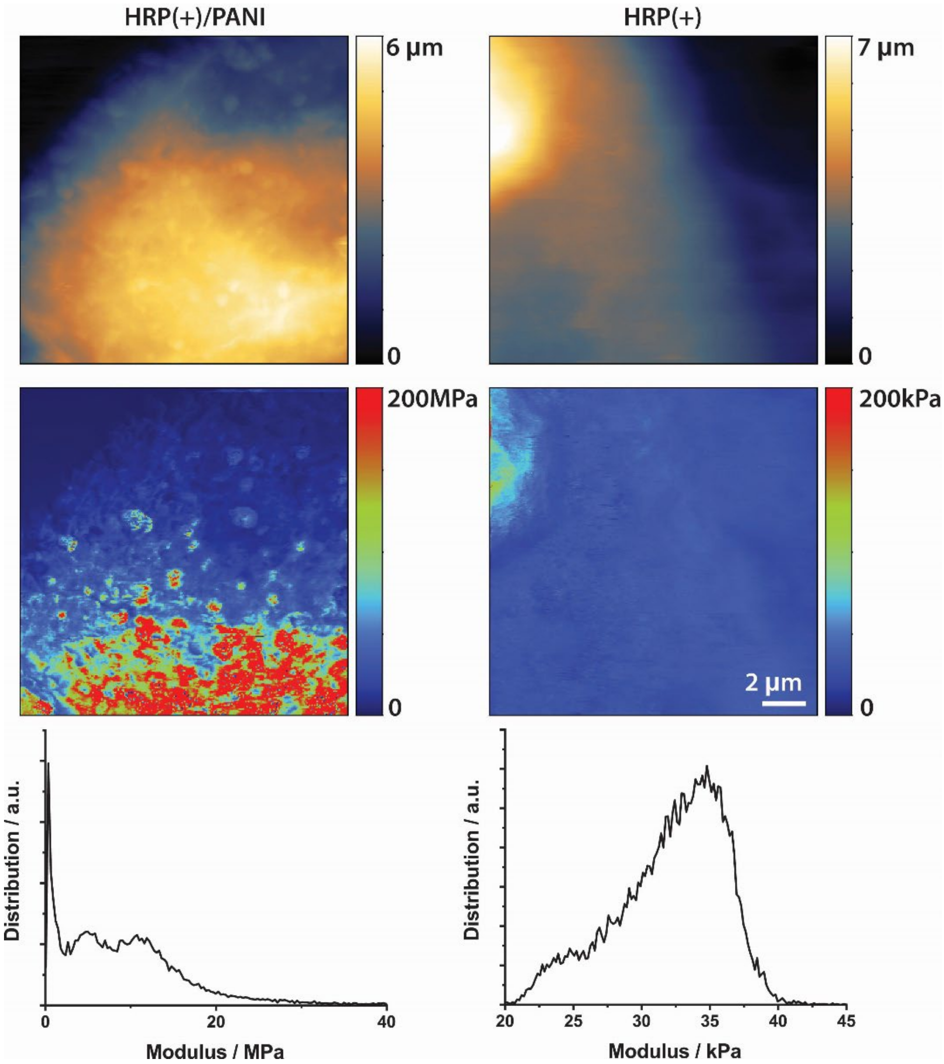

Fig. S8.

AFM images of HRP(+) neurons with and without PANI deposition. Top, original AFM height and modulus images of the panels in Fig. 4B. Bottom, distribution of the modulus in the original images.

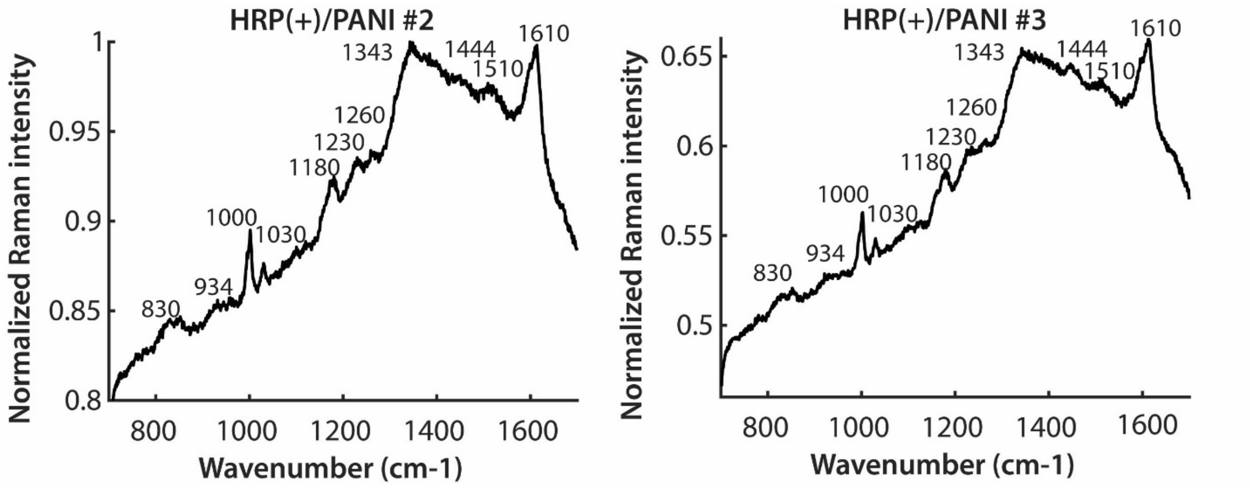

Fig. S9.

Additional Raman spectroscopy data of HRP(+)/PANI cells. This data complementing Fig. 5C for HRP(+)/PANI cell#1. #1-#3 were measured from cells on N = 3 coverslips.

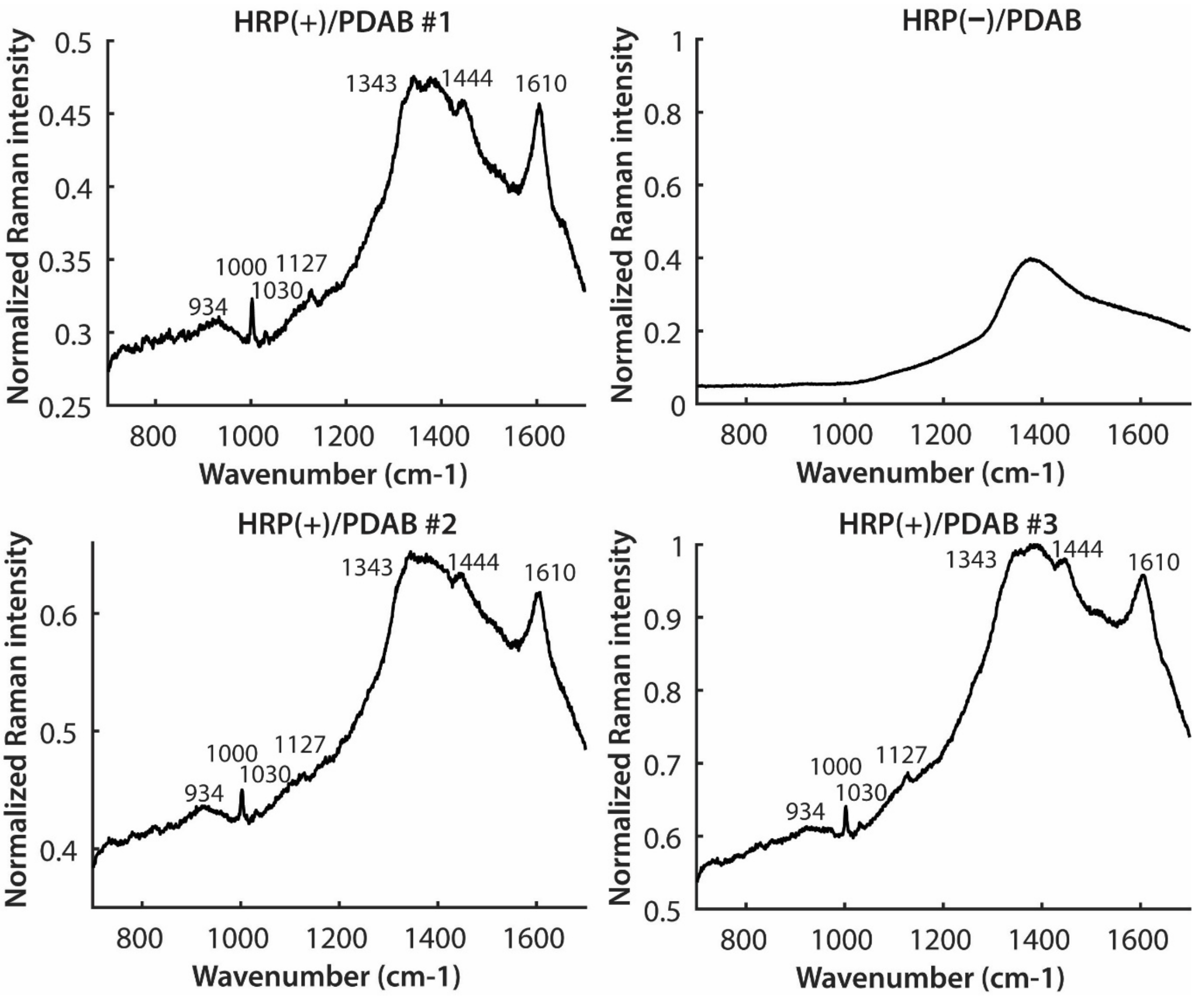

Fig. S10.

Raman spectroscopy data of HRP(+)/PDAB and HRP(−)/PDAB cells. #1-#3 were measured from cells on N = 3 coverslips.

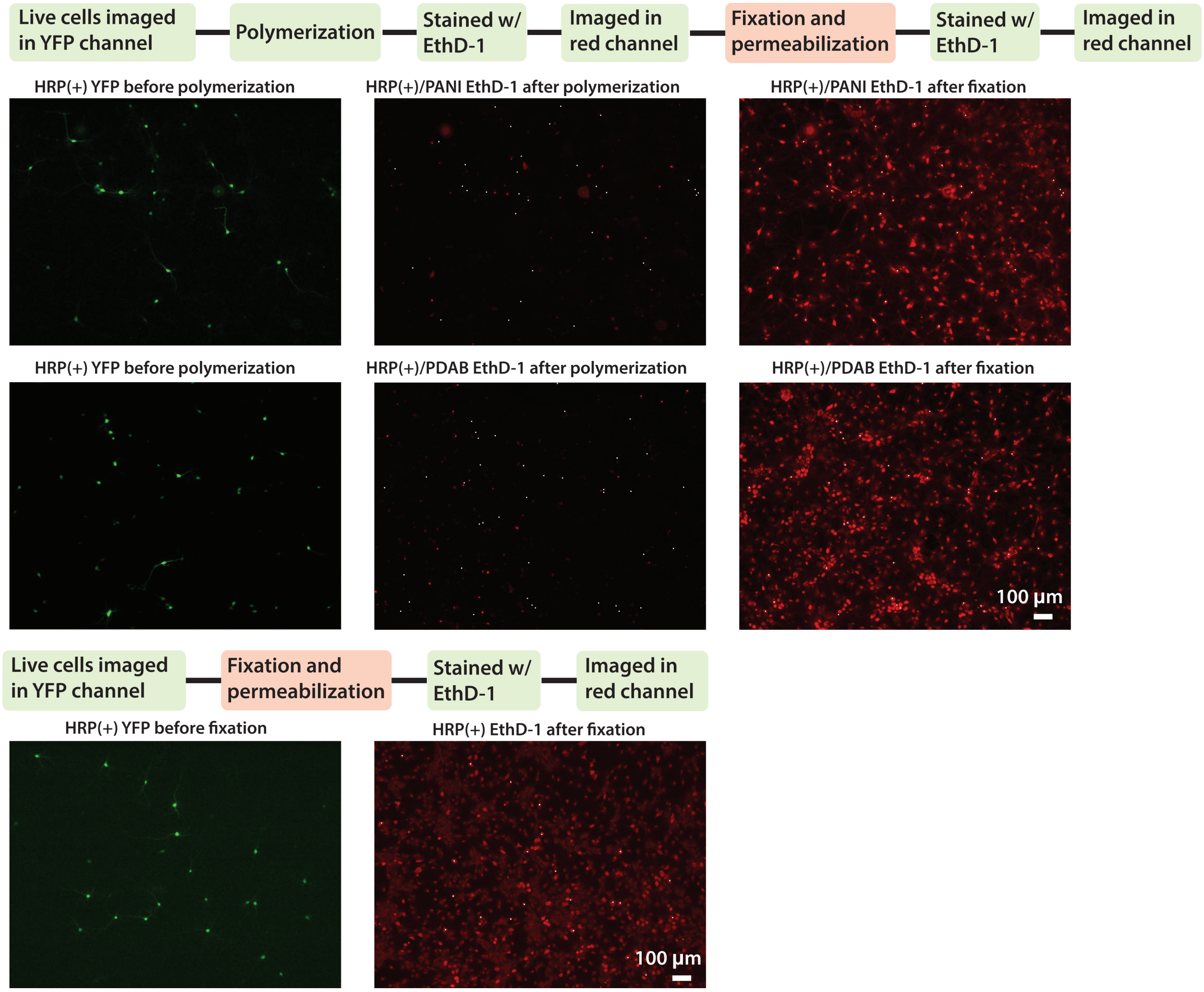

Fig. S11.

Positive control for cell viability testing. Top, live HRP(+) cells were imaged with YFP, and after PANI or PDAB deposition, were stained and imaged with ethidium homodimer (EthD-1), a red fluorophore that selectively stains dead cells with damaged membranes. Cells were then fixed and stained with EthD-1 again. White dots in the EthD-1 images mark locations of the HRP(+) cells; lack of overlay with red cells (middle) revealed that HRP(+) neurons remain viable after polymerization. White dot overlay with red cells (right) indicates that EthD-1 properly labeled dead cells. Bottom, live HRP(+) cells were imaged with YFP (left), and after fixation and permeabilization were stained with EthD-1 (right). White dot overlay with red cells indicates that EthD-1 properly labeled dead cells.

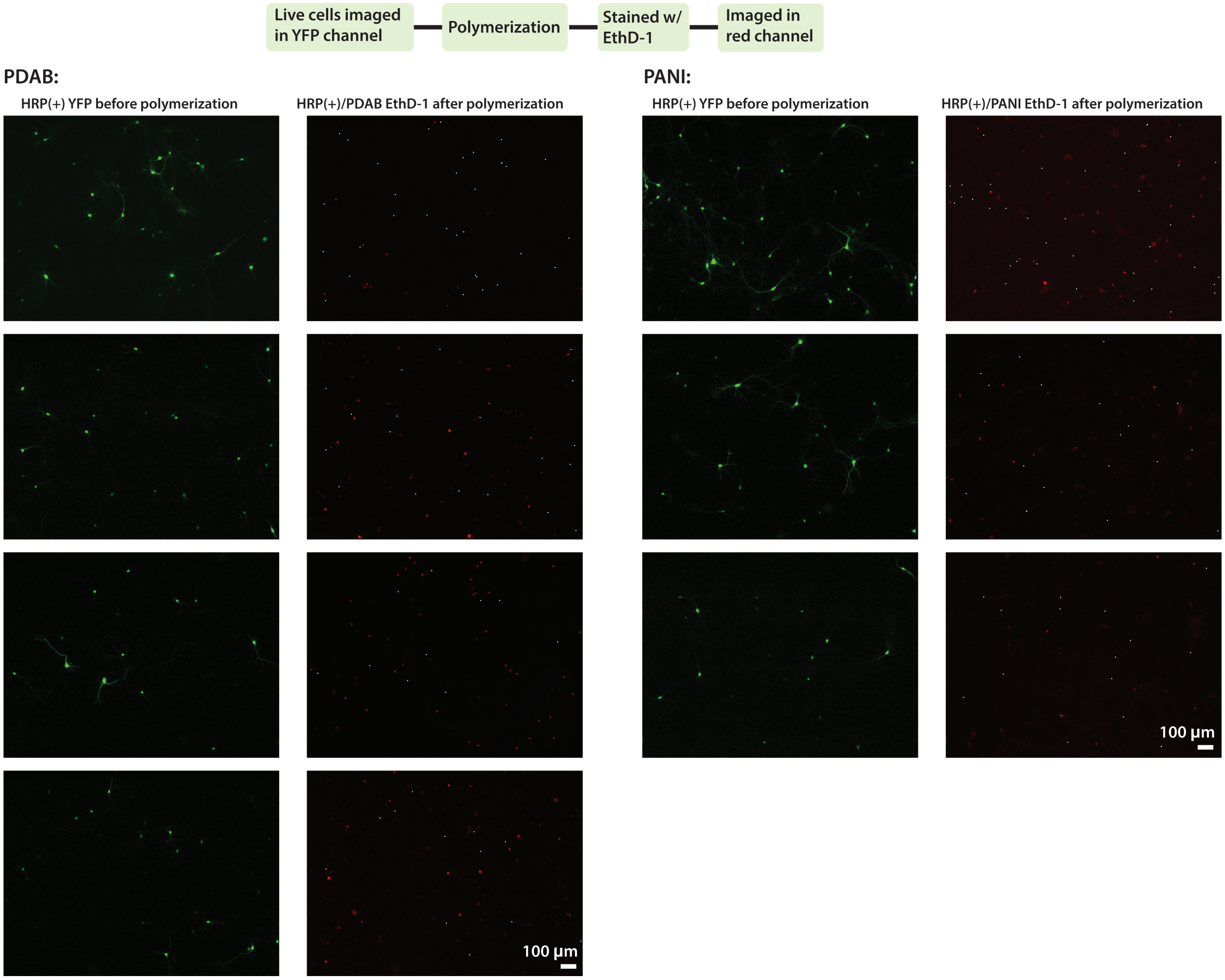

Fig. S12.

Additional viability tests of HRP(+) cells after PDAB and PANI deposition. N = 5 coverslips were used for each polymerization condition with one field of view imaged per coverslip. HRP(+) neurons remained viable after polymerization.

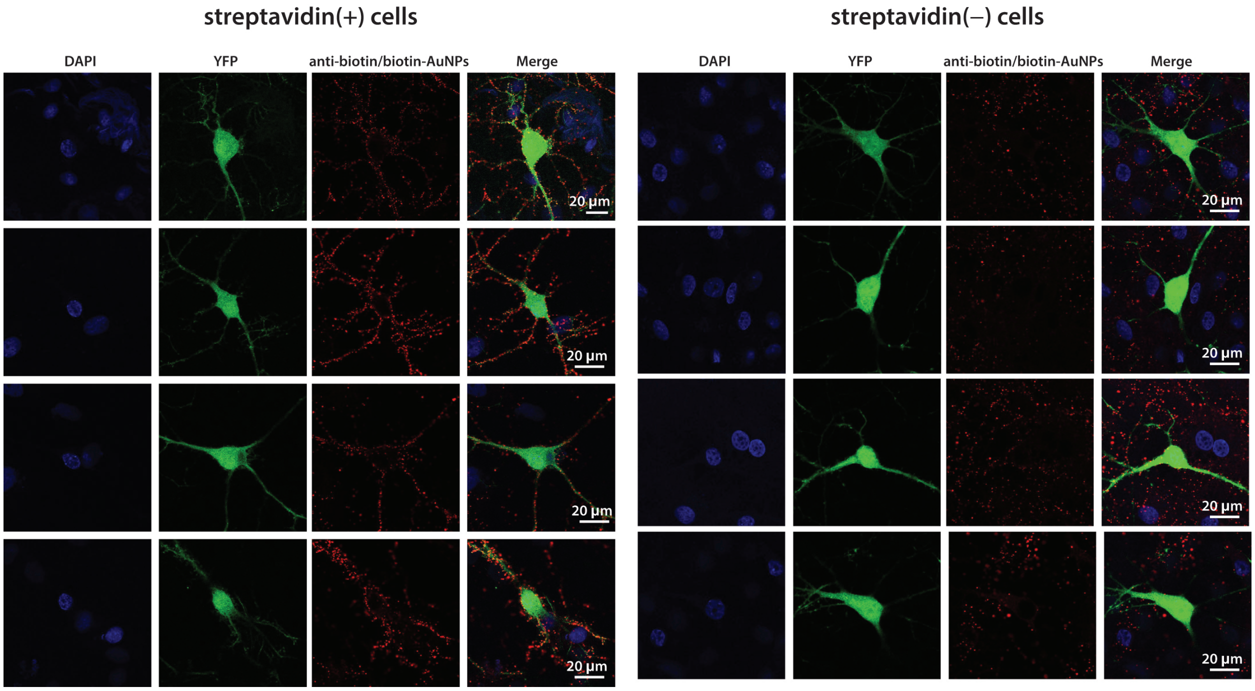

Fig. S13.

Additional results showing selective binding of biotin-Au NPs on streptavidin(+) neurons. The panels show biotin-staining results of streptavidin(+) (left) and streptavidin(−) (right) neurons after biotin-conjugated Au NP incubation. Au NPs specifically bind to streptavidin(+) neurons (complementing Fig. 6G).
